# Quantization of linear acoustic and elastic wave models in characterizations of isomorphism

**DOI:** 10.1038/s41598-024-57092-0

**Published:** 2024-04-16

**Authors:** Chen Yang

**Affiliations:** Faculty of Science and Engineering, The University of Manchester, Manchester, M13 9PL UK

**Keywords:** Physics, Quantum physics

## Abstract

From the macroscopic to the microscopic world, quantum mechanical effects in acoustics and elastic waves have become increasingly important. Observations on the quantum effects of acoustic and elastic waves using experimental methods have been reported in the literature. However, the conventional formulations of acoustic and elastic waves are still mainly governed by classical models. In this study, we investigated the quantization of acoustic and elastic waves using generalized Lorenz gauges. The potential variables of acoustic and elastic waves can be quantized in a manner similar to that of electrodynamics. The results include the Schrödinger equation with minimal coupling between the field and particles. The quantization of field variables is established as a consequence of the gauge symmetry property of the Schrödinger equation. Later, we explored the connections between the parallel formulations of mechanics and waves through an algebraic aspect. This highlights the isomorphism pattern from the theoretical characterization within the parallel formulations. To support the results, the derivations of potential formulations based on Lorenz gauges and functional mapping between field variables are presented.

## Introduction

Historically, mechanical waves, such as acoustics and elasticity, have been considered as waves that carry classical properties only. During the last few decades, continuous research and studies on acoustic and elastic media in microscopic worlds have demonstrated the important quantum mechanical properties of waves in propagation and interaction^1,2^. Recent experimental studies on small devices (e.g., thin-film beams and plates) have revealed quantum features in microscale and nanoscale mechanical devices^3–7^. In such mechanical devices, acoustic and elastic potentials are important sources of energy to interact with the external microscopic world. These experiments not only established the direction of further study on the quantum mechanics of the macroscopic world, but also demonstrated the important connections between classical and quantum worlds. In parallel, the discoveries from many experiments have generated theoretical interest in characterizing the quantum properties in acoustics and elasticity.

In the past, the foundations of acoustics and elastics were established using continuum mechanics in the classical regime. The governing equations of acoustic and elastic waves are derived from an infinitesimal element based on the equation of motion. Simultaneously, a model of electromagnetism has been developed for both field strength and gauge-potential formulations^8,9^. Unlike electromagnetism, the formulation of acoustics and elasticity is frequently given in their field-strength form. Gauge-potential formulations, such as those similar to classical electrodynamics, have not been extensively investigated. In the potential formulation of electrodynamics, Lorenz and Coulomb gauges are frequently introduced to establish field equations of electromagnetic potentials that are parallel to the formulation of field strengths^10^. Compared with other gauge approaches, the discovery of the Lorenz gauge provides a symmetrical treatment of the scalar and vector potentials in the decoupled form^10,11^.

Earlier studies on the theoretical connections between mechanical and electromagnetism involved the formulation of a classical electromagnetic model^12^. Subsequently, analogies between the gauge formulation of electrodynamics and elasticity have been studied by various authors. A symmetric relationship between the Coulomb gauge and linear elasticity was shown in^13^. The authors also discussed possible mapping between the two models based on analogous formulations^14^. Analogies between the gauge formulation of electrodynamics and acoustics are reported in^15^. Furthermore, similarities between the field strength formulation of electromagnetism and Lamé formulation of elasticity were reported in^16,17^. Although analogies between mechanical waves and electromagnetic waves have been reported in the literature, their connection with quantum properties has not been revealed. Therefore, the motivation of this study is to establish quantization of models for linear acoustic and elastic waves from the original classical forms. To achieve this, we aim to further discuss isomorphic patterns in the theoretical characterizations of parallel models within classical and quantum frameworks.

## Results

### Gauge-potential formulation of models

To begin with the quantization of waves, we chose to establish the original models via an alternative gauge-potential formulation to maximize the similarity with electrodynamics. To this end, the concept of the Lorenz gauge is generalized to waves in a medium, and it preserves the causality and gauge invariance of waves.

#### Potential formulation of acoustic waves

Based on the electromagnetism procedures presented in the Methods section, we introduced the generalized Lorenz gauge to establish the potential formulation of the waves. The original field variables of the acoustic waves are governed by inhomogeneous wave equations with external sources ${f}_{a}$ and ${{\varvec{g}}}_{a}$^18^:

1 $${\nabla }^{2}P-\frac{1}{{{\varvec{c}}}_{a}^{2}}{\partial }_{t}^{2}P={f}_{a}({\varvec{r}},t), {\nabla }^{2}{\varvec{u}}-\frac{1}{{{\varvec{c}}}_{a}^{2}}{\partial }_{t}^{2}{\varvec{u}}={{\varvec{g}}}_{a}({\varvec{r}},t),$$

where $P$ is the acoustic pressure, ${\varvec{u}}$ is the particle velocity, and ${{\varvec{c}}}_{a}$ is the acoustic velocity defined by the material properties.

2 $${{\varvec{c}}}_{a}=\sqrt{B/\rho }$$

where $B$ is the bulk modulus of the fluid and $\rho$ is the fluid density. In Eq. (2), the material properties were assumed to be constant in the acoustic medium. Next, an acoustic gauge function ${\Lambda }_{a}$ is introduced to satisfy the following wave-like gauge condition:

3 $$\nabla \cdot \left(\rho {\varvec{u}}\right)+\frac{1}{{{\varvec{c}}}_{a}^{2}}{\partial }_{t}P=0.$$

The acoustic pressure and particle velocity between different configurations can be expressed by the acoustic gauge function as follows:

4 $$\Delta P={P}{\prime}-P=-{\partial }_{t}{\Lambda }_{a}, \Delta {\varvec{u}}={{\varvec{u}}}^{\boldsymbol{^{\prime}}}-{\varvec{u}}=\frac{1}{\rho }\nabla {\Lambda }_{a},$$

where $\Delta P$ denotes the change in acoustic pressure and $\Delta {\varvec{u}}$ denotes the change in the particle velocity at different configurations . By substituting Eqs. (4) into (3), we obtained the following equation for the acoustic gauge function:

5 $${\nabla }^{2}{\Lambda }_{a}-\frac{1}{{{\varvec{c}}}_{a}^{2}}{\partial }_{t}^{2}{\Lambda }_{a}=0.$$

It can be observed that Eqs. (4) and (5) are similar to the Lorenz gauge formulation in the text on electrodynamics^10^. The gauge invariance of the above gauge potential formulation of acoustic waves can be observed. To demonstrate this feature, the body force and fluid vorticity were expressed by the field potentials.

6 $${{\varvec{f}}}_{a}=\nabla P-{\partial }_{t}(\rho {\varvec{u}}) ;{{\varvec{\omega}}}_{a}=\nabla \times {\varvec{u}},$$

where ${{\varvec{f}}}_{a}$ is the force density of the acoustic medium and ${{\varvec{\omega}}}_{a}$ is the vorticity of the acoustic medium. The above fields are not altered by the change in field potentials from old to new configurations ($P\to P{\prime},{\varvec{u}}\to {\varvec{u}}{\prime}$), as follows:

7 $${{\varvec{f}}}_{a}=\nabla {P}{\prime}-{\partial }_{t}\left(\rho {{\varvec{u}}}^{\boldsymbol{^{\prime}}}\right)=\nabla P-{\partial }_{t}(\rho {\varvec{u}}) ;{{\varvec{\omega}}}_{a}=\nabla \times {{\varvec{u}}}^{\boldsymbol{^{\prime}}}=\nabla \times {\varvec{u}}.$$

Thus, the acoustic body force and material velocity were invariant under the gauge transformations in Eq. (4).

#### Potential formulation of elastic waves

In a solid medium, the time evolution of mechanical disturbance is described by the elastic displacement wave motion from an external source^24,25^. For general three-dimensional linear elastic solids, different modes of elastic waves have been introduced in the literature. Longitudinal and transverse waves are the most common modes in elastic solids, and typically refer to tensile and shear waves in solid mechanics. Surface waves such as Rayleigh and Lamb waves also exist in solid membranes and plates. Here, we mainly consider longitudinal and transverse waves in a three-dimensional solid medium. For a longitudinal wave, the tensile stress $\sigma$ and particle velocity ${\varvec{w}}$ are governed by inhomogeneous wave equations with external sources ${f}_{l}$ and ${{\varvec{g}}}_{l}$,

8 $${\nabla }^{2}\sigma -\frac{1}{{{\varvec{c}}}_{l}^{2}}{\partial }_{t}^{2}\sigma ={f}_{l}({\varvec{r}},t), {\nabla }^{2}{\varvec{w}}-\frac{1}{{{\varvec{c}}}_{l}^{2}}{\partial }_{t}^{2}{\varvec{w}}={{\varvec{g}}}_{l}({\varvec{r}},t)$$

and

9 $${{\varvec{c}}}_{l}=\sqrt{E/\rho },$$

where $E$ is Young’s modulus and $\rho$ is the mass density of the solid. The longitudinal gauge function ${\Lambda }_{l}$ satisfying the wave-like condition can be expressed as follows:

10 $$\nabla \cdot (\rho {\varvec{w}})+\frac{1}{{{\varvec{c}}}_{l}^{2}}{\partial }_{t}\sigma =0,$$

where the tensile stress and longitudinal velocity between different states can be expressed by:

11 $$\Delta \sigma =\sigma {\prime}-\sigma =-{\partial }_{t}{\Lambda }_{l}, \Delta {\varvec{w}}={\varvec{w}}\boldsymbol{^{\prime}}-{\varvec{w}}=\frac{1}{\rho }\nabla {\Lambda }_{l},$$

where $\Delta \sigma$ denotes the change in tensile stress and $\Delta {\varvec{w}}$ denotes the change in particle velocity at different configurations. By substituting Eqs. (11) into (10) yields the following equation for the longitudinal gauge of tensile deformation:

12 $${\nabla }^{2}{\Lambda }_{l}-\frac{1}{{{\varvec{c}}}_{l}^{2}}{\partial }_{t}^{2}{\Lambda }_{l}=0.$$

For transverse waves, the shear stress $\tau$ and particle velocity ${\varvec{\gamma}}$ are governed by inhomogeneous wave equations with external sources ${f}_{t}$ and ${{\varvec{g}}}_{t}$,

13 $${\nabla }^{2}\tau -\frac{1}{{{\varvec{c}}}_{t}^{2}}{\partial }_{t}^{2}\tau ={f}_{t}({\varvec{r}},t), {\nabla }^{2}{\varvec{\gamma}}-\frac{1}{{{\varvec{c}}}_{t}^{2}}{\partial }_{t}^{2}{\varvec{\gamma}}={{\varvec{g}}}_{t}({\varvec{r}},t)$$

and

14 $${{\varvec{c}}}_{t}=\sqrt{G/\rho },$$

where $G$ is the shear modulus, and $\rho$ is the mass density. The transverse gauge function ${\Lambda }_{t}$ that satisfies the wave-like condition is expressed as follows:

15 $$\nabla \cdot (\rho{\varvec{\gamma}})+\frac{1}{{{\varvec{c}}}_{t}^{2}}{\partial }_{t}\tau =0,$$

where the shear stress and transverse velocity between different configurations can be expressed by:

16 $$\Delta \tau =\tau {\prime}-\tau =-{\partial }_{t}{\Lambda }_{t}, \Delta{\varvec{\gamma}}={\varvec{\gamma}}\boldsymbol{^{\prime}}-{\varvec{\gamma}}=\frac{1}{\rho }\nabla {\Lambda }_{t},$$

where $\Delta \tau$ denotes the change in shear stress and $\Delta{\varvec{\gamma}}$ denotes the change in particle velocity. By substituting Eqs. (16) into (15) yields the following equation for the transverse gauge of shear deformation:

17 $${\nabla }^{2}{\Lambda }_{t}-\frac{1}{{{\varvec{c}}}_{t}^{2}}{\partial }_{t}^{2}{\Lambda }_{t}=0.$$

A gauge invariance feature was established. The force density and strain vorticity are invariant under the change in elastic potentials from old to new configurations ($\sigma \to \sigma {\prime},{\varvec{w}}\to {\varvec{w}}{\prime}$),

18 $${{\varvec{f}}}_{l}=\nabla {\sigma }{\prime}-{\partial }_{t}\left(\rho {{\varvec{w}}}^{\boldsymbol{^{\prime}}}\right)=\nabla \sigma -{\partial }_{t}(\rho {\varvec{w}}) ;{{\varvec{\omega}}}_{l}=\nabla \times {{\varvec{w}}}^{\boldsymbol{^{\prime}}}=\nabla \times {\varvec{w}}$$

where ${{\varvec{f}}}_{l}$ is the force density of the elastic medium and ${{\varvec{\omega}}}_{l}$ is the vorticity of the elastic medium in the longitudinal mode. For the transverse mode, the gauge invariance of the body force and strain vorticity between the old and new configurations ($\tau \to \tau {\prime},{\varvec{\gamma}}\to{\varvec{\gamma}}{\prime}$) can be expressed as follows:

19 $${{\varvec{f}}}_{t}=\nabla {\tau }{\prime}-{\partial }_{t}\left(\rho {{\varvec{\gamma}}}^{\boldsymbol{^{\prime}}}\right)=\nabla \tau -{\partial }_{t}\left(\rho{\varvec{\gamma}}\right);{{\varvec{\omega}}}_{t}=\nabla \times {{\varvec{\gamma}}}^{\boldsymbol{^{\prime}}}=\nabla \times{\varvec{\gamma}}$$

where ${{\varvec{f}}}_{t}$ is the force density of the elastic medium and ${{\varvec{\omega}}}_{t}$ is the vorticity of the elastic medium. The gauge-potential formulation of acoustic and elastic waves manifests gauge invariance and causality properties, similar to electromagnetism. The establishment of a gauge potential formulation is the first step in the formal quantization of mechanical waves.

### Functional maps and minimal coupling

With the established gauge potential formulations, the isomorphism between the gauge and potentials in similar differential operators was studied. This leads to a functional map (relations) between the wave variables in acoustic, elasticity, and electromagnetism. The minimal coupling between the mechanics and waves was obtained from the mapping of the Lagrangian.

#### Functional map relations of field potentials

**Figure 1:**
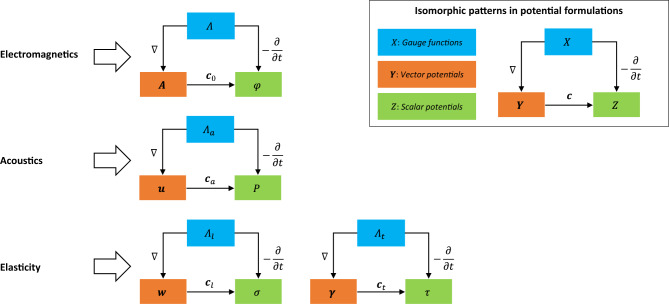
Common differential structures between wave variables in classical models.

The wave variables in the gauge-potential formulations are presented in Table 1 based on their gauge functions, field potentials, and differential equations. From the table, it can be seen that the main differences between the given potential formulations are related to the velocity parameters in the wave equations. Furthermore, because of the governing wave equations of the gauge function of the same type and field potentials are defined by the same differential operators, the isomorphic patterns between the potential formulations can be represented by the plot in Fig. 1. In the above figure, the variables are classified using three color codes to demonstrate their roles in each model. The gauge functions are shown in blue, vector potentials in orange, and scalar potentials in green. Arrow relations refer to differential or algebraic operations between variables. The isomorphic patterns are labeled in the top-right corner.

**Table 1:** Wave variables in the electromagnetic, acoustic and elastic fields.

Field models	Wave variables		Gauge-potential formulations	
Electrodynamics (Transverse)	$\Lambda =\Lambda ({\varvec{r}},t)$	$\varphi =\varphi ({\varvec{r}},t)$	${\nabla }^{2}\Lambda -\frac{1}{{\mathbf{c}}_{0}^{2}}{\partial }_{t}^{2}\Lambda =0$	$\Delta \varphi =-{\partial }_{t}\Lambda$
		${\varvec{A}}={\varvec{A}}({\varvec{r}},t)$		$\Delta {\varvec{A}}=\nabla\Lambda$
Acoustics (Longitudinal)	${\Lambda }_{a}={\Lambda }_{a}({\varvec{r}},t)$	$P=P({\varvec{r}},t)$	${\nabla }^{2}{\Lambda }_{a}-\frac{1}{{{\varvec{c}}}_{a}^{2}}{\partial }_{t}^{2}{\Lambda }_{a}=0$	$\Delta P=-{\partial }_{t}{\Lambda }_{a}$
		${\varvec{u}}={\varvec{u}}({\varvec{r}},t)$		$\Delta {\varvec{u}}=\frac{1}{\rho }\nabla {\Lambda }_{a}$
Elasticity (Longitudinal and transverse)	${\Lambda }_{l}={\Lambda }_{l}({\varvec{r}},t)$	$\sigma =\sigma ({\varvec{r}},t)$	${\nabla }^{2}{\Lambda }_{l}-\frac{1}{{{\varvec{c}}}_{l}^{2}}{\partial }_{t}^{2}{\Lambda }_{l}=0$	$\Delta \sigma =-{\partial }_{t}{\Lambda }_{l}$
		${\varvec{w}}={\varvec{w}}({\varvec{r}},t)$		$\Delta {\varvec{w}}=\frac{1}{\rho }\nabla {\Lambda }_{t}$
	${\Lambda }_{t}={\Lambda }_{t}({\varvec{r}},t)$	$\tau =\tau ({\varvec{r}},t)$	${\nabla }^{2}{\Lambda }_{t}-\frac{1}{{{\varvec{c}}}_{t}^{2}}{\partial }_{t}^{2}{\Lambda }_{t}=0$	$\Delta \tau =-{\partial }_{t}{\Lambda }_{t}$
		${\varvec{\gamma}}={\varvec{\gamma}}({\varvec{r}},t)$		$\Delta{\varvec{\gamma}}=\frac{1}{\rho }\nabla {\Lambda }_{t}$

Moreover, from the homogenous wave equations of the gauge function, the basic solution of the gauge functions is in a plain waveform:

20 $$X({\varvec{r}},t)={X}_{0}{e}^{i\theta },$$

and

21 $${\varvec{Y}}=\nabla X;Z=-{\partial }_{t}X$$

where $X$ denotes the gauge function, ${X}_{0}$ denotes the amplitude of the gauge, and ${e}^{i\theta }$ denotes the gauge phase. From the properties of the complex exponentials, the basis solution of a certain gauge function can be used to generate the basis solution of another gauge function by modifying the amplitude and phase as follows:

22 $${X}{\prime}\left({\varvec{r}},t\right)=(A{X}_{0})({e}^{i\theta }{e}^{i\alpha })={X}_{0}{\prime}{e}^{i{\theta }{\prime}}\Rightarrow {X}{\prime}\left({\varvec{r}},t\right)=\mathcal{F}\circ X\left({\varvec{r}},t\right)=\mathcal{F}(X)$$

where ${X}{\prime}$ denotes the new gauge function, $A$ denotes certain constants, ${e}^{i{\theta }{\prime}}$ denotes the phase difference, and $\mathcal{F}$ refers to the functional relation (one-to-one map) between the old gauge function $X$ and new gauge function ${X}{\prime}$. As shown in Fig. 1, the differential operations between the gauge and potential were the same for the three field models. Consequently, the functional relations between vector-like potentials can be derived using the chain rule:

23 $${{\varvec{Y}}}{\prime}=\nabla {X}{\prime}=\nabla \left(\mathcal{F}\circ X\right)=\mathcal{F}\circ \left(\nabla X\right)=\mathcal{F}\circ {\varvec{Y}}=\mathcal{F}({\varvec{Y}})$$

where ${{\varvec{Y}}}{\prime}$ and ${\varvec{Y}}$ denote the two vector-like potentials in different field models. Similarly, the functional relations between the scale-like potentials can be derived using the chain rule:

24 $${Z}{\prime}=-{\partial }_{t}{X}{\prime}=-{\partial }_{t}\left(\mathcal{F}\circ X\right)=\mathcal{F}\circ \left(-{\partial }_{t}X\right)=\mathcal{F}\circ Z=\mathcal{F}(Z)$$

From the results of Eqs. (22)–(24), the gauge functions and field potentials in different potential formulations can be connected by functional relationships. From the linearity of the differential operators, the functional maps between one set of wave variables and another can be stated as follows:

25 $$X\to ^{{\mathcal{F}}} X^{\prime}; {\varvec{Y}}\to ^{{\mathcal{F}}} \user2{Y^{\prime}}; Z\to ^{{\mathcal{F}}} Z^{\prime}.$$

#### Minimal coupling with particle mechanics

To consider the coupling between the particles and waves, the first step is the construction of the total Lagrangian, which constitutes both kinematic and potential contributions. Given the functional mapping between the wave variables, the modification of the Lagrangian can be derived by representing the electromagnetic potentials using the acoustic potentials:

26 $${\mathcal{F}}_{a}\left(\rho {\varvec{u}}\right)=q{\varvec{A}} ;{\mathcal{F}}_{a}\left(P\right)=q\varphi ,$$

where ${\mathcal{F}}_{a}$ denotes the functional relation, which gives the Lagrangian of the forced particle in terms of the acoustic potentials $P$ and ${\varvec{u}}$,

27 $$L({\varvec{v}},\varphi ,{\varvec{A}})\stackrel{{\mathcal{F}}_{a}}{\to }L({\varvec{v}},P,{\varvec{u}})=\frac{1}{2}m{{\varvec{v}}}^{2}+{\mathcal{F}}_{a}\left(\rho {\varvec{u}}\right)\cdot {\varvec{v}}-{\mathcal{F}}_{a}\left(P\right),$$

For elasticity, the functional relations between the potential variables from electromagnetism and elasticity can be applied to construct the corresponding Lagrangian of particles for the longitudinal and transverse modes, respectively.

28 $${\mathcal{F}}_{l}\left(\rho {\varvec{w}}\right)=q{\varvec{A}} ;{\mathcal{F}}_{l}\left(\sigma \right)=q\varphi ,$$

and

29 $${\mathcal{F}}_{t}\left(\rho{\varvec{\gamma}}\right)=q{\varvec{A}} ;{\mathcal{F}}_{t}\left(\tau \right)=q\varphi ,$$

where ${\mathcal{F}}_{l}$ and ${\mathcal{F}}_{t}$ denote the functional relationships of the longitudinal and transverse modes, respectively. For the longitudinal mode, the Lagrangian of the particle becomes,

30 $$L({\varvec{v}},\varphi ,{\varvec{A}})\stackrel{{\mathcal{F}}_{l}}{\to }L({\varvec{v}},\sigma ,{\varvec{w}})=\frac{1}{2}m{{\varvec{v}}}^{2}+{\mathcal{F}}_{l}\left(\rho {\varvec{w}}\right)\cdot {\varvec{v}}-{\mathcal{F}}_{l}\left(\sigma \right),$$

For transverse mode, the Lagrangian of the particle becomes,

31 $$L({\varvec{v}},\varphi ,{\varvec{A}})\stackrel{{\mathcal{F}}_{t}}{\to }L({\varvec{v}},\tau ,{\varvec{\gamma}})=\frac{1}{2}m{{\varvec{v}}}^{2}+{\mathcal{F}}_{t}\left(\rho{\varvec{\gamma}}\right)\cdot {\varvec{v}}-{\mathcal{F}}_{t}\left(\tau \right),$$

From the above Lagrangian of the particle, the total Lagrangian of the particle coupled with acoustic and elastic waves can be obtained as

32 $$L\left({\varvec{v}},P,{\varvec{u}},\sigma ,{\varvec{w}},\tau ,{\varvec{\gamma}}\right)=\frac{1}{2}m{{\varvec{v}}}^{2}+\left[{\mathcal{F}}_{a}\left(\rho {\varvec{u}}\right)+{\mathcal{F}}_{l}\left(\rho {\varvec{w}}\right)+{\mathcal{F}}_{t}\left(\rho{\varvec{\gamma}}\right)\right]\cdot {\varvec{v}}-\left[{\mathcal{F}}_{a}\left(P\right)+{\mathcal{F}}_{l}\left(\sigma \right)+{\mathcal{F}}_{t}\left(\tau \right)\right].$$

The Hamiltonian of the particle can be obtained by the Legendre transform as follows:

33 $$H\left({\varvec{p}},P,{\varvec{u}},\sigma ,{\varvec{w}},\tau ,{\varvec{\gamma}}\right)=\frac{1}{2m}{\left[{\varvec{p}}-{\mathcal{F}}_{a}\left(\rho {\varvec{u}}\right)-{\mathcal{F}}_{l}\left(\rho {\varvec{w}}\right)-{\mathcal{F}}_{t}\left(\rho{\varvec{\gamma}}\right)\right]}^{2}+\left[{\mathcal{F}}_{a}\left(P\right)+{\mathcal{F}}_{l}\left(\sigma \right)+{\mathcal{F}}_{t}\left(\tau \right)\right].$$

Therefore, the Hamiltonian of free particle ${H}_{0}$ can be rewritten as

34 $${H}_{0}=H-\left[{\mathcal{F}}_{a}\left(P\right)+{\mathcal{F}}_{l}\left(\sigma \right)+{\mathcal{F}}_{t}\left(\tau \right)\right],$$

and the momentum of free particle ${{\varvec{p}}}_{0}$ can be rewritten as:

35 $${{\varvec{p}}}_{0}={\varvec{p}}-{\mathcal{F}}_{a}\left(\rho {\varvec{u}}\right)-{\mathcal{F}}_{l}\left(\rho {\varvec{w}}\right)-{\mathcal{F}}_{t}\left(\rho{\varvec{\gamma}}\right),$$

The above two equations show the minimal coupling between the particle and mechanical waves in the classical framework. The quantum properties are not explicitly accessible from the above relations. Nevertheless, with the connections between the mechanical and electromagnetic waves, the quantization of the acoustic and elastic waves will share procedures in the quantization of the electromagnetic waves.

### Schrödinger equation and wave quantization

From the dynamic variables and minimal coupling, the quantum equation of mechanics and waves is established from the modification of the dynamic operators. The fixing of the action magnitude using the Planck constant uniquely determines the Schrödinger equation. The gauge symmetry of the Schrödinger equation provides sufficient conditions for quantization of the wave variables.

#### Schrödinger equation of particle with waves

For a free particle, the conservation laws of the Hamiltonian and momentum lead to a homogenous wave equation of action, which leads to wave-like dynamic variables^26,27^,

36 $${\nabla }^{2}S-\frac{1}{{{\varvec{v}}}^{2}}{\partial }_{t}^{2}S=0\Rightarrow S({\varvec{r}},t)=-i{S}_{0}{\phi }_{0} ,$$

where ${S}_{0}$ denotes the amplitude of the action and ${\phi }_{0}$ denotes the phase function of the free particle. From the linear differential operator, the Hamiltonian of the particle can be represented by the differential operators of the phase function as

37 $${H}_{0}=i{S}_{0}{\partial }_{t}{\phi }_{0}\Rightarrow {\widehat{H}}_{0}=i{S}_{0}{\partial }_{t} ,$$

and the momentum can be represented by the operator form,

38 $${{\varvec{p}}}_{0}=-i{S}_{0}\nabla {\phi }_{0} \Rightarrow {\widehat{{\varvec{p}}}}_{0}=-i{S}_{0}\nabla .$$

From the minimal coupling with the mechanical waves in Eqs. (34) and (35) yield the following relationship for differential operators:

39 $$H={H}_{0}+\Delta H=i{S}_{0}{\partial }_{t}{\phi }_{0}+\Delta H=i{S}_{0}{\partial }_{t}\left({\phi }_{0}+\Delta \phi \right)=i{S}_{0}{\partial }_{t}\phi \Rightarrow \widehat{H}=i{S}_{0}{\partial }_{t} ,$$

where $\Delta H$ denotes the modification of the Hamiltonian, $\Delta \phi$ denotes an additional term for the phase function, and $\phi$ denotes the phase function of the particle. Similarly, the momentum of a particle in minimal coupling with the field potentials can be obtained using Eqs. (44) and (47), respectively.

40 $${\varvec{p}}={{\varvec{p}}}_{0}+\Delta {\varvec{p}}=-i{S}_{0}\nabla {\phi }_{0}+\Delta {\varvec{p}}=-i{S}_{0}\nabla \left({\phi }_{0}+\Delta \phi \right)=-i{S}_{0}\nabla \phi \Rightarrow \widehat{{\varvec{p}}}=-i{S}_{0}\nabla ,$$

where $\Delta {\varvec{p}}$ denotes modification of momentum. By resolving the action amplitude via the Planck constant ${S}_{0}\to \hslash$ and replacing the phase function with the wave function $\phi \to \psi$, the Hamiltonian and momentum of the particle arrive at the usual expression in quantum mechanics^28^. By substituting the differential operators in Eq. (33), the following Schrödinger equation of the particle and minimal coupling with acoustic and elastic waves are obtained:

41 $$i\hslash {\partial }_{t}\psi =\left(\frac{1}{2m}{\left[-i\hslash \nabla -{\mathcal{F}}_{a}\left(\rho {\varvec{u}}\right)-{\mathcal{F}}_{l}\left(\rho {\varvec{w}}\right)-{\mathcal{F}}_{t}\left(\rho{\varvec{\gamma}}\right)\right]}^{2}+\left[{\mathcal{F}}_{a}\left(P\right)+{\mathcal{F}}_{l}\left(\sigma \right)+{\mathcal{F}}_{t}\left(\tau \right)\right]\right)\psi .$$

The result in Eq. (41) is the quantum–mechanical version of the classical equation in Eq. (33). In the above equation, the particle is characterized by dynamic operators associated with the wave function, whereas the classical fields are characterized by mechanical wave variables.

#### Gauge symmetry and wave quantization

Gauge symmetry of Schrödinger equation is well-known for particle in electromagnetism^30^. From the functional relations wave variables in Eq. (25), the gauge symmetry properties can be shown for the acoustics and elasticity. For acoustics, the transformation of the wave functions ($\psi \to \psi {\prime}$) by the acoustic gauge function does not change the Schrödinger equation in Eq. (41):

42 $${\psi }{\prime}={e}^{i\lambda \left({\varvec{r}},t\right)}\psi ={e}^{i{\mathcal{F}}_{a}\left({\lambda }_{a}\left({\varvec{r}},t\right)\right)}\psi ; {\lambda }_{a}\left({\varvec{r}},t\right)={\mathcal{F}}_{a}^{-1}\left(\lambda \right)={\mathcal{F}}_{a}^{-1}\left(\frac{q}{\hslash }\Lambda \right)=\frac{q}{\hslash }{\Lambda }_{a},$$

where ${\mathcal{F}}_{a}^{-1}$ is the inverse functional map between the acoustic and electromagnetic potentials, which fulfills the unity condition ${\mathcal{F}}_{a}^\circ {\mathcal{F}}_{a}^{-1}=1$. Similarly, for elasticity, the gauge symmetry of the Schrödinger equation can be found with the following transformations based on elastic gauge functions from the functional relations:

43 $${\psi }{\prime}={e}^{i\lambda \left({\varvec{r}},t\right)}\psi ={e}^{i{\mathcal{F}}_{l}\left({\lambda }_{l}\left({\varvec{r}},t\right)\right)}\psi ; {\lambda }_{l}\left({\varvec{r}},t\right)={\mathcal{F}}_{l}^{-1}\left(\lambda \right)={\mathcal{F}}_{l}^{-1}\left(\frac{q}{\hslash }\Lambda \right)=\frac{q}{\hslash }{\Lambda }_{l}$$

and

44 $${\psi }{\prime}={e}^{i\lambda \left({\varvec{r}},t\right)}\psi ={e}^{i{\mathcal{F}}_{t}\left({\lambda }_{t}\left({\varvec{r}},t\right)\right)}\psi ; {\lambda }_{t}\left({\varvec{r}},t\right)={\mathcal{F}}_{t}^{-1}\left(\lambda \right)={\mathcal{F}}_{t}^{-1}\left(\frac{q}{\hslash }\Lambda \right)=\frac{q}{\hslash }{\Lambda }_{t}.$$

where ${\mathcal{F}}_{l}^{-1}$ and ${\mathcal{F}}_{t}^{-1}$ are the inverse functional maps between the elastic and electromagnetic potentials, and they fulfill the unity condition ${\mathcal{F}}_{l}^\circ {\mathcal{F}}_{l}^{-1}={\mathcal{F}}_{t}^\circ {\mathcal{F}}_{t}^{-1}=1$. Because of the gauge functions are additive, the symmetry of Eq. (41) can be ensured by linear superposition of the individual gauge functions:

45 $${\psi }{\prime}={e}^{i\sum \lambda \left({\varvec{r}},t\right)}\psi ; \sum \lambda =\lambda +{\mathcal{F}}_{a}({\lambda }_{a})+{\mathcal{F}}_{l}({\lambda }_{l})+{\mathcal{F}}_{t}({\lambda }_{t})=\frac{q}{\hslash }\left(\Lambda +{\Lambda }_{a}+{\Lambda }_{l}+{\Lambda }_{t}\right).$$

The gauge symmetries of the Schrödinger equation from acoustics and elasticity show gauge field features that are similar to those of electromagnetism as a linear superposition.

The gauge symmetry of particle in quantum mechanics poses the constraint on gauge function and field potentials. In electromagnetism, it introduces the quantum properties of scalar and vector potentials from gauge function that fulfills the gauge symmetry. In acoustic wave variables, from Eq. (42) and by applying Eq. (4) which yields,

46 $$P=-{\partial }_{t}{\mathcal{F}}_{a}^{-1}\left(\lambda \right)\frac{\hslash }{q}={\mathcal{F}}_{a}^{-1}\left(\hslash \Delta \omega {\lambda }_{a}\right) ; \rho {\varvec{u}}=\nabla {\mathcal{F}}_{a}^{-1}\left(\Lambda \right)\frac{\hslash }{q}={\mathcal{F}}_{a}^{-1}(\hslash \Delta {\varvec{k}}{\lambda }_{a}).$$

Similarly, in elastic wave variables, from Eqs. (43) and (11) it gives the follow:

47 $$\sigma =-{\partial }_{t}{\mathcal{F}}_{l}^{-1}\left(\lambda \right)\frac{\hslash }{q}={\mathcal{F}}_{l}^{-1}\left(\hslash \Delta \omega {\lambda }_{l}\right) ; \rho {\varvec{w}}=\nabla {\mathcal{F}}_{l}^{-1}\left(\Lambda \right)\frac{\hslash }{q}={\mathcal{F}}_{l}^{-1}(\hslash \Delta {\varvec{k}}{\lambda }_{l}),$$

and from Eqs. (44) and (16) it gives the follow:

48 $$\tau =-{\partial }_{t}{\mathcal{F}}_{t}^{-1}\left(\lambda \right)\frac{\hslash }{q}={\mathcal{F}}_{t}^{-1}\left(\hslash \Delta \omega {\lambda }_{t}\right) ; \rho{\varvec{\gamma}}=\nabla {\mathcal{F}}_{t}^{-1}\left(\Lambda \right)\frac{\hslash }{q}={\mathcal{F}}_{t}^{-1}(\hslash \Delta {\varvec{k}}{\lambda }_{t}).$$

As a result of gauge symmetry in quantum mechanics, the permissible energy and momentum of wave variables in acoustic and elasticity are included from the inverse functional maps. The relations in Eqs. (46)–(48) bridge the classical properties and quantum properties of the mechanical wave variables in linear acoustic and elastic models.

### Some potential applications

From a practical aspect, quantization of the classical acoustics and elastic waves can be applied to provide quantum description of certain classical phenomenon. This closely bridges the wave-like picture in classical regime and particle-like picture in quantum regime. Furthermore, it provides a comprehensive model of particle-beam interaction with both wave-like components and particle-like components.

#### Radiation beam with opaque object

One of the examples could be the radiation pressure which is widely established in modern optics where the radiation pressure can be described by the energy–momentum exchange of opaque object. From the functional mapping between acoustic and electromagnetism, the quantum (particle-like) picture of the radiation pressure from acoustic or elastic wave beam can be formulated. Considering the acoustic beam radiated from a point source, the resultant force of acoustic beam on an opaque object is given by,

49 $${{\varvec{F}}}_{rad}=\int {P}_{rad}ds=\int \left[{\mathcal{F}}_{a}^{-1}\left(\hslash \Delta \omega {\lambda }_{a}\right)\right]ds ,$$

where ${\varvec{F}}$ is acoustic radiation force, $s$ denotes the applied surface and Eq. (46) is applied into above equation for acoustic pressure. From the relation of wave parameter, the radiant momentum can be represented by the volume integral of momentum density,

50 $${p}_{rad}=\int {{\varvec{F}}}_{rad}dt=\iint \left[{\mathcal{F}}_{a}^{-1}\left(\hslash \Delta \omega {\lambda }_{a}\right)\right]dsdt=\iint \left[{\mathcal{F}}_{a}^{-1}\left(\hslash \Delta {\varvec{k}}{\lambda }_{a}\right)\right]dsd{\varvec{l}}=\iiint \left[{\mathcal{F}}_{a}^{-1}\left(\hslash \Delta {\varvec{k}}{\lambda }_{a}\right)\right]dV,$$

where is the $d{\varvec{l}}$ infinitesimal length that wave passing through during time increment $dt$ and $dV$ is the infinitesimal volume. By dividing the total radiant momentum by individual term, the above equation can be given in the discretized form based on infinitesimal approximation,

51 $${p}_{rad}=\sum d{p}_{rad}\cong \sum \left[{\mathcal{F}}_{a}^{-1}\left(\hslash \Delta {\varvec{k}}{\lambda }_{a}\right)\right]dV \Rightarrow d{p}_{rad}={\mathcal{F}}_{a}^{-1}\left(\hslash \Delta {\varvec{k}}{\lambda }_{a}\right)dV,$$

The above equation links the radiation momentum density with quantum parameters of acoustic beam interacting with opaque object.

#### Interaction with waves and particles

A further extension of the above example is the scenarios with waves and particles that are interacted in the same spatial domain of interest. Frequently, these scenarios include complex time evolution behavior that part of the problem is modeled as continuous propagated waves while the other part is represented as discretized tracing particles. During the area of interaction, the exchange of energy–momentum occurs that can be described by the relation in Eq. (41). For instance, before and after interaction with linear acoustic waves, the dynamics of particle can be represented by:

52 $$i\hslash {\partial }_{t}\psi =\left(\frac{1}{2m}{\left[-i\hslash \nabla -{\mathcal{F}}_{a}\left(\rho {\varvec{u}}\right)\right]}^{2}+{\mathcal{F}}_{a}\left(P\right)\right)\psi \Rightarrow i\hslash {\partial }_{t}\psi {\prime}=\frac{1}{2m}{\left(-i\hslash \nabla \right)}^{2}\psi {\prime}.$$

where $\psi$ and $\psi {\prime}$ denotes the wave function before and after interaction, respectively. The conservation of energy and momentum between particle and waves are the given by the following:

53 $$i\hslash {\partial }_{t}\psi +{\mathcal{F}}_{a}\left(P\right)=H+{\mathcal{F}}_{a}\left(P\right)={H}{\prime}+{\mathcal{F}}_{a}\left({P}{\prime}\right)=i\hslash {\partial }_{t}{\psi }{\prime}+{\mathcal{F}}_{a}\left({P}{\prime}\right)$$

and

54 $$-i\hslash \nabla \psi +{\mathcal{F}}_{a}\left(\rho {\varvec{u}}\right)={\varvec{p}}+{\mathcal{F}}_{a}\left(\rho {\varvec{u}}\right)={{\varvec{p}}}{\prime}-{\mathcal{F}}_{a}\left(\rho {{\varvec{u}}}^{\boldsymbol{^{\prime}}}\right)=-i\hslash \nabla {\psi }{\prime}+{\mathcal{F}}_{a}\left(\rho {{\varvec{u}}}^{\boldsymbol{^{\prime}}}\right)$$

where $P{\prime}$ and ${\varvec{u}}{\prime}$ denotes the acoustic pressure and material velocity after the interaction with particle. The new wave variables of acoustic pressure and material velocity are derived from new gauge function ${\Lambda }_{a}{\prime}$ via relation in Eq. (4). At the same time, the dynamics of propagated acoustic waves can be descripted as the following,

55 $${\nabla }^{2}{\Lambda }_{a}-\frac{1}{{{\varvec{c}}}_{a}^{2}}{\partial }_{t}^{2}{\Lambda }_{a}=0 \Rightarrow {\nabla }^{2}{\Lambda }_{a}{\prime}-\frac{1}{{{\varvec{c}}}_{a}^{2}}{\partial }_{t}^{2}{\Lambda }_{a}{\prime}=0.$$

The above example illustrates the dynamics of particles and waves before and after the exchange of their energy and momentum in the same spatial region.

## Discussion

The presented quantization of linear acoustic and elastic waves carries two levels of information. At the first level, the theoretical formulation of linear waves extends the original models in the classical regime and yields a consistent result in the quantum regime. It bridges the classical and quantum properties of wave variables of linearized acoustics and elasticity. The transition of the wave variables between the classical regime and quantum regime is governed by the angular frequency and wave vector. The increase in the angular frequency of the excitation of small objects in the microscopic world enhances the quantum effect. This makes the quantum effect relatively visible compared to the large-sized object in the macroscopic world. In contrast, the decrease in the angular frequency and wave vector in the wave pattern weakens the quantum effect and approximates the usual classical description.

**Figure 2:**
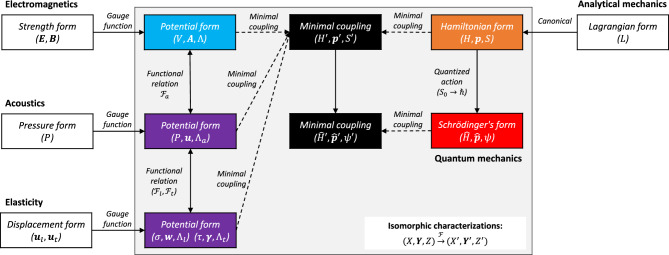
Isomorphic characterizations of mechanics and waves in classical and quantum regimes.

To provide a picture of the different parallel formulations, their connections are categorized and shown in Fig. 2. The different connected formulations were labeled with color, and the rest were labeled as white. In detail, the gauge-potential formulation of electromagnetism is labeled blue. The gauge-potential formulations of acoustics and elasticity are labeled purple. The Hamiltonian and quantum formulations of the particle dynamics are labeled orange and red, respectively. The solid arrows indicate the key procedures that change one formulation into another. The dashed arrows refer to the couplings between the potentials with dynamic variables between the field and particles. The shaded box in the figure shows the key similarities and differences between the characterizations of the models. In particular, the isomorphic patterns in characterizing electromagnetism, acoustics, elasticity, and particle dynamics in elementary models are shown.

Moreover, at the second level, the isomorphism on the theoretical characterizations of these elementary models provide a common algebraic view. In this view, the above models can be represented as vector spaces that are equipped with one-to-one linear mappings^29^. Since the configuration of vector space ($X, {\varvec{Y}}, Z$) for each model is characterized via the associated physical variables, the linear mappings between the configuration in vector spaces are represented by the functional map relations between the dynamic and wave variables. From this perspective, the coupling between these models can be formally represented by the linear superposition of individual vector spaces in a certain sequence. From Fig. 2, the resultant model is represented as a vector space (${X}_{n}, {{\varvec{Y}}}_{n}, {Z}_{n}$) that constitutes the contribution of each elementary model, such as,

56 $$\begin{gathered} X_{n} = X_{0} + X_{e} + X_{a} + X_{l} + X_{t} ; {\varvec{Y}} = {\varvec{Y}}_{0} + {\varvec{Y}}_{e} + {\varvec{Y}}_{a} + {\varvec{Y}}_{l} + {\varvec{Y}}_{t} ; \hfill \\ Z = Z_{0} + Z_{e} + Z_{a} + Z_{l} + Z_{t} , \hfill \\ \end{gathered}$$

where the subscript label $e,a,l,t$ denotes the configurations in the models of electromagnetism, acoustic and elasticity (longitudinal and transverse). The resulting vector space is dimensionally preserved because the vector space of every element model has the same dimension (rank). The entire permissible vector spaces that can be represented by the linear superposition of the elementary models from a set of vector spaces that include the three types of particle-wave coupling and the three types of wave-wave couplings.

## Methods

### Gauge-potential formulation of electromagnetism

In classical electromagnetism^10^, by introducing the Lorenz gauge function $\Lambda$, the scalar and vector potentials are governed by inhomogeneous wave equations with non-vanishing charge density $\varrho$ and current density ${\varvec{j}}$:

57 $${\nabla }^{2}\varphi -\frac{1}{{{\varvec{c}}}_{0}^{2}}{\partial }_{t}^{2}\varphi =f(\varrho ), {\nabla }^{2}{\varvec{A}}-\frac{1}{{{\varvec{c}}}_{0}^{2}}{\partial }_{t}^{2}{\varvec{A}}={\varvec{g}}({\varvec{j}}),$$

where $\varphi$ is the scalar potential, ${\varvec{A}}$ is the vector potential, and ${{\varvec{c}}}_{0}$ is the speed of light in vacuum, which is defined based on electromagnetic constants:

58 $${{\varvec{c}}}_{0}=\frac{1}{\sqrt{{\epsilon }_{0}{\mu }_{0}}}$$

where ${\epsilon }_{0}$ is the vacuum permittivity, and ${\mu }_{0}$ is the magnetic permeability. The Lorenz gauge function satisfies the following gauge condition:

59 $$\nabla \cdot {\varvec{A}}+\frac{1}{{{\varvec{c}}}_{0}^{2}}{\partial }_{t}\varphi =0$$

and the field potentials between different configurations can be expressed by:

60 $$\Delta \varphi ={\varphi }{\prime}-\varphi =-{\partial }_{t}\Lambda , \Delta {\varvec{A}}={{\varvec{A}}}^{\boldsymbol{^{\prime}}}-{\varvec{A}}=\nabla\Lambda ,$$

where $\Delta {\varvec{V}}$ denotes the change in the scalar potential and $\Delta {\varvec{A}}$ denotes the change in the vector potential at different configurations. As in^10^, from Eqs. (59) and (60), it can be shown that the Lorenz gauge function satisfies the following equation:

61 $${\nabla }^{2}\Lambda -\frac{1}{{{\varvec{c}}}_{0}^{2}}{\partial }_{t}^{2}\Lambda =0.$$

Other gauge functions, such as the Coulomb gauge (quasi-static) or general velocity gauge (extension of the Lorenz gauge), have also been reported in the literature. It is worth noting from the literature that the technical procedures for arriving at gauge equations are not unique and parallel methods have been studied and discussed by the authors^19–21^. A comprehensive summary of the different gauge conditions and their relationships can be found in the literature^22,23^. The Coulomb gauge leads to instantaneous propagation of the vector potential. The velocity gauge leads to different velocities between the propagation of scalar and vector potentials.

### Gauge invariance of electrodynamics

For a given gauge function and applying the gauge transform, the pair of field potentials is changed from old to new configurations. Recalling the gauge transform in electromagnetism, the field strength and field potentials are bridged by the following relations:

62 $${\varvec{E}}=\nabla \varphi -{\partial }_{t}{\varvec{A}} ;{\varvec{B}}=\nabla \times {\varvec{A}},$$

where ${\varvec{E}}$ is the electrical field, and ${\varvec{B}}$ is the magnetic field. The above fields were not altered by the change in field potentials from old to new configurations ($\varphi \to {\varphi }{\prime},{\varvec{A}}\to {\varvec{A}}{\prime}$):

63 $${\varvec{E}}=\nabla \varphi {\prime}-{\partial }_{t}{\varvec{A}}\boldsymbol{^{\prime}}=\nabla \varphi -{\partial }_{t}{\varvec{A}} ;{\varvec{B}}=\nabla \times {\varvec{A}}\boldsymbol{^{\prime}}=\nabla \times {\varvec{A}}.$$

This shows gauge invariance in the potential formulation of classical electromagnetism, where the field strength and field force are unaltered before and after the gauge transform.

As in the potential formulation of electrodynamics^10^, the corresponding Lagrangian of the charged particle in the electromagnetic field is given by:

64 $$L({\varvec{v}},\varphi ,{\varvec{A}})=\frac{1}{2}m{{\varvec{v}}}^{2}+q{\varvec{A}}\cdot {\varvec{v}}-q\varphi ,$$

where $q$ is the unit of charge. The coupling between the particle and electromagnetic field is reflected from the potential-related terms in the Lagrangian. If the electromagnetic potential vanishes identically, the Lagrangian of the particle is reduced to that of a free particle in classical mechanics. Furthermore, the Lorentz force can be directly derived from the Lagrangian of the particle in the potential form as

65 $${\varvec{F}}=q\left[\left(-\nabla \left(\varphi -{\varvec{v}}\cdot {\varvec{A}}\right)\right)-\frac{d{\varvec{A}}}{dt}\right],$$

The example above shows the equivalent Lagrangian of a particle with its external force in the potential formulation. Moreover, from the Lagrangian, the Hamiltonian of the particle can be obtained by the Legendre transform, as in classical mechanics, which yields:

66 $$H({\varvec{p}},\varphi ,{\varvec{A}})=\frac{1}{2m}{\left({\varvec{p}}-q{\varvec{A}}\right)}^{2}+q\varphi ,$$

In field theory, the results of Eq. (34) refer to the minimal coupling (the simplest case without the spin effect) between the electromagnetic field and the charged particle. The interaction between the field and particle can include higher-order multipole moments of the charge to reflect the spin effect, as in Pauli coupling^30^.

### Gauge symmetry of wave function

Gauge symmetry is a fundamental property of the gauge field theory that provides a description of the particle and field in quantum mechanics. By recalling the gauge symmetry from electromagnetism, the gauge symmetry of acoustics and elasticity can be demonstrated using functional relations. For electromagnetism, the Schrödinger equation remains unaltered when the following transformations of the wave functions from old to new configurations are performed by the phase function ($\psi \to \psi {\prime}$):

67 $${\psi }{\prime}={e}^{i\lambda \left({\varvec{r}},t\right)}\psi ; \lambda \left({\varvec{r}},t\right)=\frac{q}{\hslash }\Lambda \left({\varvec{r}},t\right),$$

where $\lambda$ denotes the phase function, which is space and time dependent. From the above relation, the scalar and vector potentials can be derived from gauge function from Eq. (60):

68 $$q\varphi =-{\partial }_{t}\Lambda =\hslash \omega \lambda \left({\varvec{r}},t\right) ;q{\varvec{A}}=\nabla\Lambda =\hslash {\varvec{k}}\lambda \left({\varvec{r}},t\right).$$

## Data Availability

All data generated or analysed during this study are included in this published article.
